# Trajectories of psychological distress and social integration in newly resettled refugees: findings from the Building a New Life in Australia longitudinal study

**DOI:** 10.1007/s00127-023-02528-7

**Published:** 2023-07-01

**Authors:** Thomas P. Nguyen, Shameran Slewa-Younan, Pilar Rioseco

**Affiliations:** 1 Mental Health, School of Medicine, Western Sydney University, Locked Bag 1797, Penrith South DC, Sydney, NSW 1797 Australia; 2 School of Medicine, Translational Health Research Institute, Western Sydney University, Sydney, NSW Australia; 3 School of Psychiatry, Academic Unit of Child Psychiatry South West Sydney, University of New South Wales, Sydney, NSW Australia; 4 Centre for Mental Health, Melbourne School of Population and Global Health, University of Melbourne, Melbourne, VIC Australia; 5 Australian Institute of Family Studies, Melbourne, VIC Australia; 6 School of Public Health and Social Work, Queensland University of Technology, Brisbane, QLD Australia

**Keywords:** Refugees, Resettlement, Psychological distress, Mental illness, Mental health

## Abstract

**Purpose:**

Refugees are at greater risk of mental illness due to stressors encountered post-resettlement. However, few longitudinal studies have examined the within-person effects of these stressors, especially with respect to social integration. This study aims to examine what factors are associated with psychological distress in a longitudinal sample of refugees resettled in Australia.

**Methods:**

This study used data from three Waves of the Building a New Life in Australia study, collected between 2013 and 2018. The eligible sample included 1881 adult respondents, clustered in 1175 households. We conducted multilevel mixed-effects growth modelling incorporating time-variant and time-invariant covariates with psychological distress, using the Kessler Psychological Distress Scale (K6).

**Results:**

Rates of high psychological distress increased across the 5-year follow-up period. Social integration stressors (e.g. discrimination, lower sense of belonging, loneliness, lower English proficiency) were associated with higher levels of psychological distress over time. Refugees reporting loneliness not only had a greater risk of elevated psychological distress at each time point, but the difference in risk increased over each time point. Refugees who were exposed to traumatic events, older, female and of Middle Eastern background were also more likely to report higher levels of psychological distress over time.

**Conclusions:**

These findings highlight the importance of identifying refugees who may encounter difficulties with social integration in the early years of resettlement. Newly arrived refugees may benefit from longer-term resettlement programmes that address post-migratory stressors, particularly with regards to loneliness, to reduce the prevalence of elevated psychological distress during the early years of resettlement.

**Supplementary Information:**

The online version contains supplementary material available at 10.1007/s00127-023-02528-7.

## Introduction

Over the last decade, refugee numbers worldwide have continued to rise, reaching an unprecedented high of 27.1 million people in 2021 [1]. Refugees forced to leave their home country due to instances such as war, violence or political persecution may be repeatedly exposed to stressors as well as potentially traumatic events (PTEs) pre- and peri-migration [2]. Repeated exposure to PTEs and torture have been shown to predict more severe mental health outcomes in the refugee population [3, 4]. Alarmingly, meta-analytic data suggest that the prevalence rate of diagnosed PTSD and depression in refugees resettled in high-income countries may be as high as 30% for both mental disorders [2]. These rates far surpass the prevalence of PTSD and depression in the general population and are a serious cause of concern for mental health clinicians and refugee resettlement services alike. Moreover, refugees with even subclinical levels of mental illness may be at elevated risk of symptom deterioration, citing the need for early intervention for those at risk [5].

Numerous factors have been associated with poorer mental health outcomes in resettled refugees. One meta-analysis found that those who were older, female, from rural regions and had a higher socioeconomic and educational background prior to resettlement reported poorer mental health outcomes, whilst mental health outcomes appeared to improve over time [6]. A prior study suggested that refugees born in the Middle East were at a higher risk of severe mental illness compared with those from other regions [7]. Despite these risk factors, refugees continue to experience adversities that predispose to developing a mental illness (e.g. prolonged separation from family, language barriers and unemployment) following resettlement in a host country [2, 8]. Thus, psychosocial interventions targeting post-resettlement factors may be considered a more effective strategy for refugees when compared to pre-settlement stressors because they are more easily managed in the short term and are more proximal predictors of current psychological distress [9]. For example, one randomized-controlled trial reported significant reductions in psychopathology following an intervention targeting stressors related to social integration [10]. In fact, a growing body of research suggests that post-resettlement factors may even be more robust predictors of mental health outcomes than pre-settlement stressors [11, 12].

With respect to socioeconomic stressors during the post-resettlement period, longitudinal data has repeatedly reported positive associations between paid employment and improvements in mental health outcomes [13–15]. Meta-analytic data has also revealed that refugees and internally displaced persons who resettle in institutional and temporary private accommodation report significantly poorer mental health outcomes than those who had found permanent, private housing [6].

Prior research looking at the role of post-migratory stressors on the mental health of refugees in high-income countries has found that social integration stressors such as loneliness [7, 16, 17], discrimination [7, 18–20], language barriers [13, 17, 21, 22], acculturative stress [23] and prolonged separation from family members following resettlement [24] have been associated with poorer mental health. Meanwhile, a greater sense of belonging [25, 26] and stronger social support from both intra-ethnic and inter-ethnic groups [7, 13, 14] have been found to be protective against mental illness. Given the strong relationship between social integration stressors and mental health outcomes, it is important to further understand what stressors increase the risk of refugees reporting high levels of psychological distress during the early years of resettlement.

### The present study

Given the difficulty to recruit and access refugee populations for research purposes, large national longitudinal studies understanding the relationship between post-migratory stressors and mental health outcomes have been scarce [27]. Moreover, existing longitudinal studies have investigated post-resettlement factors associated with mental health mainly through between-person analyses. However, little research to date has looked at individual-level longitudinal associations of social integration stressors with mental health outcomes. This enables a better understanding of individual trajectories of mental health over time and helps identify which factors could be addressed to modify those trajectories, beyond the influence of individual differences. Identifying what specific post-resettlement psychosocial factors most strongly influence the development, maintenance and remission of psychological distress in the refugee population is crucial in informing the delivery of mental health interventions for clinicians and resettlement services alike. This study used mixed-effects growth modelling, incorporating time-variant and time-invariant covariates to examine changes in psychological distress over the first 5 years of settlement among participants in the Building a New Life in Australia (BNLA) longitudinal study. It was hypothesized that refugees who reported poorer social integration stressors would have higher levels of psychological distress over time. It was also hypothesized that experiences of fewer PTEs and greater time since displacement would be associated with lower levels of psychological distress.

## Methods

### Study design

The dataset used in this study was sourced from the BNLA longitudinal study. The BNLA is a large, government-commissioned project that aimed to understand early resettlement outcomes in a national sample of humanitarian migrants who had recently received permanent protection visas in Australia [28, 29]. The study design was informed by interviews and focus groups with key stakeholders including humanitarian migrants and refugee resettlement service providers [30]. The BNLA sample included humanitarian migrants who obtained their permanent protection visa offshore (before migration) or onshore (while in Australia). Eligible participants arrived in Australia (offshore pathway) or were granted a permanent visa (onshore pathway) between May and December 2013. Data was collected from the study sample annually across five different time points (hereinafter referred to as Waves). Wave 1 data was collected between October 2013 and March 2014, whilst Wave 5 was conducted from October 2018 to March 2018 [29]. The BNLA covered a broad range of measures including English language proficiency, education and employment, immigration experience, mental health, community support and perceptions of life in Australia. An overview of all survey measures available has been previously detailed elsewhere [30].

The BNLA study was approved by the Australian Institute of Family Studies Human Research Ethics Committee (protocol 13/03). We gained approval from the Department of Social Services to access the BNLA study dataset.

### Participants

Participants in this study include refugees (offshore pathway) aged 18 years and over. We excluded participants who were granted visas after arrival into Australia (i.e. seeking asylum after arriving in Australia), as asylum seekers experience significantly higher rates of mental illness and additional psychosocial stressors during the early resettlement period [31, 32]. These stressors include reduced access to social services, visa status insecurity and difficulties gaining work permits. From the 2399 individuals who participated in Wave 1, 1881 adult refugee respondents (aged 18 +), clustered across 1175 households were included in this study. Further details regarding the full study cohort have been previously described at length [28, 30].

### Study procedure

All potential participants were randomly selected from the Department of Social Services’ Settlement Database using 11 pre-identified study sites across Australia, including major cities and regional centres with large populations of newly resettled humanitarian migrants. Each potential participant was identified as either an individual or a representative of their family, based on the principal applicant (PA) on the visa application [30].

All randomly selected PAs were sent a translated letter and brochure regarding the study. These were followed up by either a home visit or a phone call. Respected members of local migrant communities were also employed to help recruit potential participants. All PAs gave consent to partake in the present study before other family members were invited to participate. Data from Waves 1, 3 and 5 were collected through home visits whilst phone interviews were used for Waves 2 and 4 [30]. Questionnaires and participant materials were translated into 14 languages in Wave 1, and 9 languages in subsequent Waves. Translations were undertaken by a professional translating company and professional interpreters were available for participants with additional language requirements.

### Measures

#### Outcome measure

Psychological distress was measured through the Kessler Screening Scale for Psychological Distress (K6). The K6 is a six-item scale that measures levels of psychological distress experienced in the last month (e.g. fatigue, hopelessness, and irritability) on a five-point Likert scale from ‘none of the time’ (1) to ‘all of the time’ (5). The K6 demonstrates strong psychometric properties and has been shown to be a sensitive tool for detecting changes over time following treatment [33]. It has also been validated to screen for psychiatric diagnoses across several languages and populations, including refugees [34, 35]. The K6 scale showed high reliability in the current sample (Cronbach’s alpha = 0.89 at baseline). A cutoff was used to separate the cohort between those who had low levels of psychological distress (scores 6 to 13) and those with moderate or high levels (scores 14 and over)^1^ [36].

#### Independent variables

##### Potentially traumatic events

In Wave 3, participants were asked whether they had experienced or witnessed one of nine PTEs before coming to Australia (e.g. murder, torture, kidnapping or imprisonment). There was an additional option of endorsing another PTE under the category ‘other’. Participants were also able to endorse options such as ‘does not apply’, ‘I don’t know’ or ‘prefer not to say’. Given the importance of this measure in our study, responses ‘I don’t know’ were coded as missing (*n* = 56 at Wave 3),^2^ while ‘does not apply’ and ‘prefer not to say’ were retained as valid response categories.

##### Social integration stressors

English proficiency, understanding and speaking proficiency, was self-assessed with the item ‘thinking about your English, how well do you…’. Response options ranged from ‘very well’ to ‘not at all’. The mean of understanding and speaking was obtained and reversed so that higher scores indicated better English proficiency. Exposure to discrimination was assessed with the single item ‘In the last 12 months, do you think you have been discriminated against, stopped from doing something, or been hassled or made to feel inferior, because of your ethnicity, religion or skin colour? (yes/no). Loneliness and acculturative stress were collected with the following question: ‘Have any of the following been a source of stress in your life in the last 12 months?’ The response options included ‘Loneliness (e.g. homesick, lack of social life and/or friends)’ and ‘getting used to life in Australia’. Respondents who selected these options were classified as experiencing loneliness and acculturative stress, respectively. Sense of belonging was assessed with the question ‘Do you feel part of the Australian community’. Response options ranged from 1 ‘always’ to 5 ‘never’. Responses were reversed and used as a continuous variable in the analysis, with higher values indicating higher sense of belonging. Finally, respondents were asked ‘Do you still have family in another country waiting to come to Australia?’ (yes/no). There variables were measured at Waves 1, 3 and 5 and were time variant in the analysis.

##### Socioeconomic stressors

Socioeconomic stressors included employment, housing and financial hardship. Individuals were classified within an ‘unemployed household’ if no household members reported being in paid work in the past 7 days. Financial hardship was measured with the question ‘In the last 12 months, has any of the following happened to you because you didn’t have enough money?’ Six financial hardship items were collected, including not being able to pay bills on time, not being able to pay rent/mortgage on time and going without meals. The number of financial hardships experiences, from 0 to 6, was used in the analysis. The socioeconomic stressors were measured at Waves 1, 3 and 5 and were used as time variant in the analysis.

##### Sociodemographic characteristics

At Wave 1, age, gender, country of birth, current location, length of stay in Australia and pre-migration education level were collected from participants. For the purposes of this study, we used country of birth as a proxy for nationality. Length of stay in Australia was categorized as either ‘less than 6 months’ or ‘6 months or more’ prior to the time of the first interview. Prior education levels were coded as either ‘never attended school’, ‘6 + years of schooling’ or ‘post-school qualification’.

### Statistical analysis

Multilevel mixed-effects logistic models were used to examine the likelihood of respondents showing elevated psychological distress (level 2, between-person differences), as well as trajectories of elevated psychological distress over time (level 1, within-person effects). These models allowed for the use of all available cases, including those with missing values [37]. The mixed model included fixed effects, the sample average, and random effects, individual variation around the sample average [38]. Only face-to-face Waves of data collection were used in this analysis (Waves 1, 3 and 5) as there was evidence of mode effects. That is, K6 scores were lower when data was collected via telephone, compared to the scores for the same individuals when data was collected face to face (results available on request). Mixed-effects models focus on changes over time; therefore, time-varying measures were examined for the predictors of interest, and the effect of change in these measures on the outcome variable was estimated. Bivariate associations as well as fully adjusted models are presented. Sensitivity analysis was also conducted excluding the variable on PTE from the models. This question was included in Wave 3; therefore, participants who did not complete a Wave 3 interview were missing in this variable. All analyses were performed on Stata 17.

## Results

In total, 4035 individuals or families were identified as potential participants for the BNLA study [39]. Of this total, 2399 individuals successfully completed Wave 1 of the study and 1,881 individuals met inclusion criteria for this study. Over 81% of eligible respondents also participated in Waves 3 and 5 (*n* = 1527 and *n* = 1531, respectively). There were no statistically significant differences in the distribution of demographic characteristics between Wave 1 and Wave 5 participants, with most variables showing variations of 1% or less. The sample comprised of 934 women (49.7%), with the sample having predominantly arrived less than 6 months before the start of Wave 1 (96.1%). Most of the sample originated from the Middle East (56.8%), mostly from Iraq and Iran, whilst all refugees from Central Asia were born in Afghanistan (23.6%) Table 1.

**Table 1:** Participant characteristics at baseline

Characteristic	*N* or mean	% or SD
Age (mean, SD)	(37.30)	(13.84)
Sex		
Male	947	50.4
Female	934	49.7
Location		
Major city	1691	89.9
Regional	190	10.1
Time since arrival		
< 6 months	1808	96.1
6–12 months	73	3.9
Region of birth		
Middle East	1068	56.8
Central Asia	444	23.6
Africa	106	5.6
South and South-East Asia	263	14.0
Pre-migration education		
Never attended school	333	17.9
6 + years of schooling	1256	67.3
Post-school qualification	275	14.8
Number of PTE		
1	422	28.7
2–3	415	28.2
4 +	368	25.0
Does not apply	169	11.5
Prefer not to say	97	6.6
Unemployed household		
Yes	1809	96.4
No	68	3.6
Housing tenure		
Temporary	157	8.5
Short term	699	37.9
Long term	957	51.9
Other	31	1.7
Number of financial hardships (mean, SD)	(0.76)	(1.13)
English proficiency (mean, SD)^a^	(1.99)	(0.78)
Experienced discrimination		
No	1801	95.9
Yes	77	4.1
Loneliness is source of stress		
No	1504	83.1
Yes	307	17.0
Waiting for family to migrate		
No	810	43.2
Yes	1067	56.9
Getting used to life in Australia (as a source of stress)		
No	1361	75.2
Yes	450	24.9
Sense of belonging (mean, SD)^b^	(4.08)	(1.06)

Notes. *PTE* potentially traumatic events. Missing values excluded from calculation of percentage. ^a^Higher number indicates better English proficiency. ^b^Higher number indicates higher sense of belonging

The percentage of adult refugees reporting moderate or high levels of psychological distress was 45.1% at Wave 1, 47.5% at Wave 3 and was lower at Wave 5 (41.3%). These proportions were similar when only including respondents who completed all three Waves of data collection. This suggests that attrition was not directly related to level of psychological distress in this sample (see Supplementary Table 1). The percentage of participants classified as experiencing elevated psychological distress at baseline, by sociodemographic characteristics, experiences of PTE, socioeconomic stressors and social integration stressors are presented in Supplementary Tables 2 and 3.

Fully adjusted models revealed a non-linear pattern of elevated psychological distress among refugees (Table 2), with a lower proportion of respondents falling into this category at Wave 1, compared with Waves 3 (OR 1.71, 95% CI [1.37, 2.14], *p* < 0.001) and 5 (OR 1.40, 95% CI [1.10, 1.78], *p* < 0.01). The proportion of respondents showing elevated levels of psychological distress was marginally higher at Wave 3 compared with Wave 5, in the adjusted models.

**Table 2:** Bivariate and adjusted mixed-effects models predicting elevated psychological distress over time

	Unadjusted model^a^			Adjusted model		
	Odds ratio	95% CI interval]	*P* > z	Odds ratio	95% CI	*P* > z
Time (ref. Wave 5, 2017/18)						
Wave 1 (2013/14)	1.27	[1.08, 1.50]	0.004	0.71	[0.56, 0.91]	0.006
Wave 3 (2015/16)	1.42	[1.20, 1.69]	< 0.001	1.22	[0.99, 1.51]	0.059
Demographic characteristics						
Age (cont.)	1.03	[1.02, 1.03]	< 0.001	1.02	[1.01, 1.02]	< 0.001
Female (ref. male)	1.70	[1.40, 2.07]	< 0.001	1.53	[1.24, 1.90]	< 0.001
Time since arrival (ref. < 6 months)						
6–12 months	0.66	[0.39, 1.09]	0.104	0.68	[0.40, 1.16]	0.158
Region of birth (ref. Middle East)						
Central Asia	0.42	[0.34, 0.53]	< 0.001	0.40	[0.29, 0.54]	< 0.001
Africa	0.28	[0.18, 0.43]	< 0.001	0.26	[0.15, 0.48]	< 0.001
South and South-East Asia	0.20	[0.15, 0.27]	< 0.001	0.35	[0.23, 0.51]	< 0.001
Education pre-migration (ref. post-school qualification)						
No schooling	0.83	[0.59, 1.17]	0.283	0.86	[0.55, 1.33]	0.486
6 + years of schooling	0.85	[0.65, 1.13]	0.269	0.98	[0.72, 1.32]	0.873
Lives in regional area (ref. major city)	0.96	[0.70, 1.31]	0.789	1.95	[1.34, 2.83]	< 0.001
Experience of PTEs pre-migration						
Number of PTEs (ref. does not apply)						
1	2.72	[1.86, 3.98]	< 0.001	1.57	[1.06, 2.33]	0.024
2–3	3.42	[2.33, 5.01]	< 0.001	1.55	[1.03, 2.31]	0.035
4 +	4.58	[3.10, 6.77]	< 0.001	1.76	[1.16, 2.67]	0.008
Prefer not to say	1.94	[1.15, 3.28]	0.013	1.54	[0.89, 2.65]	0.121
Socioeconomic stressors						
Unemployed household (ref. household member in paid work)	2.46	[2.00,3.04]	< 0.001	1.66	[1.30,2.12]	< 0.001
Housing tenure (ref. long-term housing)						
Temporary	1.14	[0.82, 1.59]	0.440	1.10	[0.76, 1.59]	0.621
Short term	0.78	[0.58, 1.06]	0.108	1.41	[1.11, 1.80]	0.005
Other	0.91	[0.54, 1.53]	0.722	1.16	[0.68, 1.98]	0.574
Number of financial hardships (cont.)	1.51	[1.41, 1.61]	< 0.001	1.39	[1.29, 1.50]	< 0.001
Social integration stressors						
English proficiency (cont.)	0.64	[0.57, 0.72]	< 0.001	0.74	[0.64, 0.86]	< 0.001
Has experienced discrimination (ref. no)	2.07	[1.49, 2.88]	< 0.001	1.64	[1.11, 2.43]	0.013
Loneliness is a source of stress (ref. no)	3.24	[2.59, 4.05]	< 0.001	2.27	[1.76, 2.93]	< 0.001
Waiting for family to migrate (ref. no)	1.29	[1.10, 1.52]	0.002	1.25	[1.04, 1.51]	0.017
Sense of belonging (cont.)	0.60	[0.55, 0.65]	< 0.001	0.63	[0.57, 0.70]	< 0.001
Getting used to life in Australia is source of stress (ref. no)	2.81	[2.27, 3.48]	< 0.001	1.75	[1.37, 2.23]	< 0.001
Constant				1.00	[0.39, 2.57]	0.999
Individual-level variance				1.38	[1.03, 1.85]	

Notes. *PTE* potentially traumatic events. *CI* confidence interval. *OR* odds ratio. *Ref* reference category. *Cont* continuous. ^a^Unadjusted models include time

**Fig. 1:**
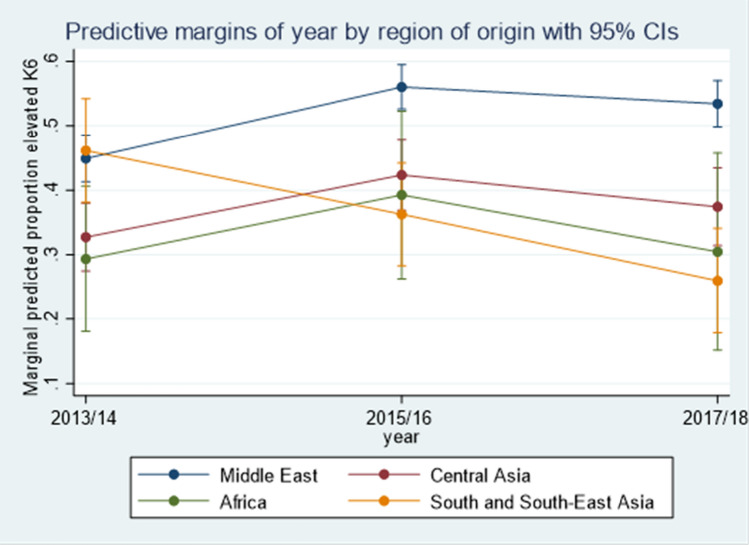
Predicted values interaction effect between region of birth and time

Demographic characteristics associated with increased risk of elevated psychological distress over time included older age, female sex and having been born in the Middle East. The largest effect size was for country of birth; the effect on the risk of elevated psychological distress for those born in the Middle East was between 2.5 (OR = 0.40, 95% CI [0.29, 0.54], *p* < 0.001) and 2.9 (OR = 0.35, 95% CI [0.23, 0.51], *p* < 0.001) times the effect for participants born in Central Asia and South and East Asia, respectively. A significant interaction effect between region of birth and time was observed, where refugees from the Middle East were consistently at higher risk of elevated psychological distress over time, compared with refugees from other regions (Fig. 1). While the pattern over time was similar between refugees from the Middle East and those from Central Asia and Africa, respondents from South and South-East Asia showed higher levels of distress at Wave 1, but a downward trend in risk of elevated psychological distress over time. Notably, for each additional year of age at migration, the odds of elevated psychological distress increased by 2% (OR = 1.02, 95% CI [1.01, 1.02], *p* < 0.001).

**Fig. 2:**
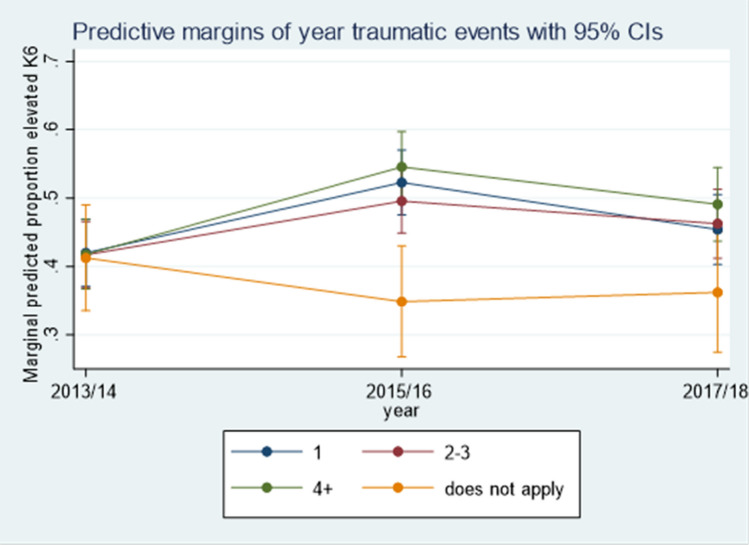
Predicted values interaction effect between potentially traumatic events and time

Refugees who experienced PTEs before migration were more likely to show elevated levels of psychological distress over time, compared to those who reported that these PTEs did not apply to them (Fig. 2). Socioeconomic stressors were significantly associated with increased risk of elevated psychological distress over the observation period. Unemployment, housing instability and financial hardship increased the risk of elevated psychological distress over time.

**Fig. 3:**
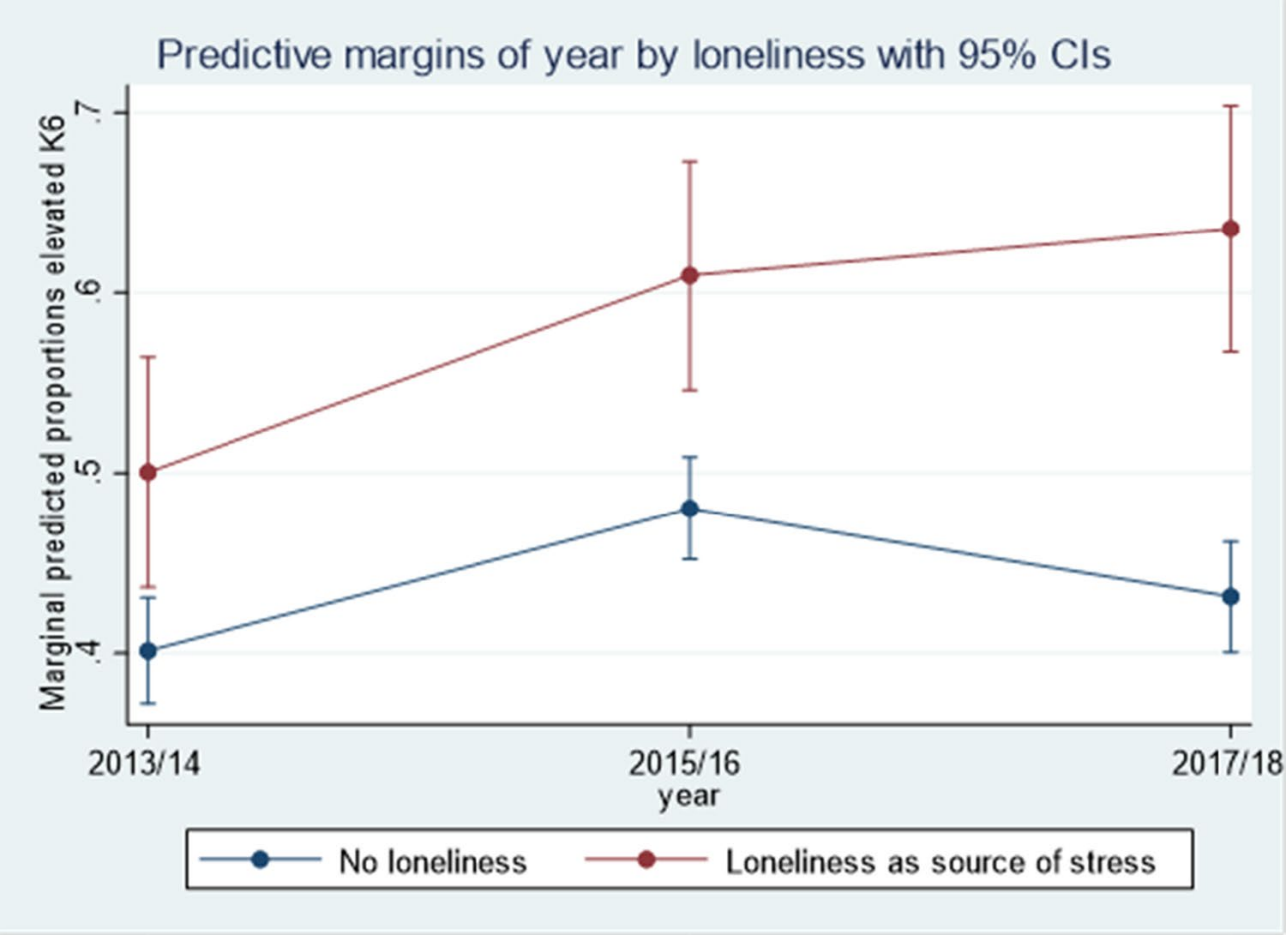
Predicted values interaction effect between loneliness and time

Social integration stressors were strongly associated with worsening in psychological distress over time. Experiencing loneliness as a source of stress was the single strongest social integration predictor of elevated psychological distress over the 5 years of the study (OR 2.27, 95% CI [1.76–2.93]). Moderation analysis revealed that the trajectories of psychological distress differed by whether refugees experienced loneliness or not. Refugees experiencing loneliness not only had a higher risk of elevated psychological distress at baseline, but also the difference in risk of elevated psychological distress increased over time. The proportion of refugees reporting elevated psychological distress increased over time among those experiencing loneliness whereas it decreased between Wave 3 and Wave 5 for those not experiencing loneliness (Fig. 3). Predicted values based on the fully adjusted model indicate that over 60% of refugees experiencing loneliness at Wave 5 reported elevated psychological distress, compared with 43% of those not experiencing loneliness.

Experiencing discrimination, poorer English proficiency as well as waiting for family to migrate to Australia, were also associated with increased risk of elevated psychological distress over time. Higher sense of belonging was a protective factor, contributing to reduced psychological distress in the early years of settlement, while experiencing difficulties getting used to life in Australia increased the risk of elevated psychological distress.

Sensitivity analysis showed that results were largely consistent with the fully adjusted model when excluding PTEs from the analysis (see Supplementary Table 4).

## Discussion

This paper sought to understand which stressors were associated with trajectories of greater psychological distress in refugees recently resettled in Australia over a 5-year follow-up period. With respect to social integration stressors, we found increased loneliness, waiting for family members and discrimination were associated with higher rates of psychological distress whilst having a greater sense of belonging and English proficiency were protective factors. We also found rates of high psychological distress increased in a non-linear pattern and were related to older age, female gender, being of Middle Eastern background, experience of PTEs and socioeconomic stress. Concerningly, the rate of individuals at moderate to high risk of psychological distress in our sample was also much higher than those in the general Australian population (7% men, 11% women) [40].

The overall trajectory of psychological distress worsened over the 5-year follow-up period, with refugees most likely to report high levels of psychological distress in Wave 3. One explanation for this finding may stem from a ‘honeymoon period’ where refugees may feel more positive about resettling into a new country at first [41]. Newly arrived refugees in Australia are able to access the Humanitarian Settlement Program for between 6 and 18 months which provides them with support in accessing accommodation, employment, English courses and links to local community groups [42]. The lack of access to resettlement services, or the transition to mainstream services, by Wave 3 may explain the higher rates of elevated psychological distress reported in our study, suggesting funding for support services should extend beyond the first 2 years of resettlement. These findings did not support our initial hypothesis or findings from a meta-analysis, which found greater time since displacement was associated with better mental health outcomes [6]. However, the aforementioned meta-analysis included studies with internally displaced populations and the mean length of displacement in the included studies ranged from 1 month to 41 years [6], whereas there was little variability in the length of stay in the current BNLA sample. Future follow-up Waves of the BNLA cohort may enable a further assessment of this trend.

We also found strong associations between most social integration stressors with a greater risk of reporting elevated levels of psychological distress over time. Experiencing loneliness was the strongest predictor amongst all stressors, with refugees experiencing loneliness reporting consistently higher rates of elevated psychological distress at each time point. Whilst prior research has shown an association between loneliness and poorer mental health outcomes in refugees [7, 16, 17], this study is the first to show that rates of high psychological distress may continue to increase in lonely refugees across the first 5 years of resettlement, whilst rates of high psychological distress decrease in those that do not experience loneliness. One previous BNLA analysis reported the association between loneliness and psychological distress fluctuated across Waves 1–4 [17]. However, we removed Waves 2 and 4 from our analyses, as interviews for those Waves were conducted over telephone and we noted that individual rates of psychological distress were much lower in those Waves when compared to those conducted in person.

Whilst it has been previously reported that Middle Eastern refugees are more likely to report higher levels of psychological distress than refugees from other regions [7], our study is the first to show that Middle Eastern refugees were consistently at a higher risk of elevated psychological distress over the first 5 years of resettlement, when compared to refugees from other regions. We hypothesize that the ongoing conflict and civil unrest in Iraq and the terror associated with Islamic State during the periods of Wave 1 and Wave 3 may have contributed to these elevated rates of psychological distress.

Our findings suggesting that respondents who are older, female and who endorse socioeconomic stressors are more likely to report elevated levels of psychological distress reinforce findings from prior research [6, 13–15]. Those who reported that PTEs did not apply to them showed a different trajectory of psychological distress over the 5-year follow-up period and were less likely to report high levels of psychological distress when compared with their baseline. The effects of pre-migration traumas clearly continue to affect levels of psychological distress in this population of refugees 5 years post-resettlement, which reflects existing findings reporting on the long-term negative effects of trauma on the mental health of refugee populations [43]. Thus, screening for trauma and providing trauma-informed care for refugees continues to be important considerations for mental health clinicians and resettlement services. Resettlement services should also provide continuous support for refugees to obtain secure housing tenure and job opportunities to prevent financial hardship and the psychological distress associated with it.

This study has several limitations that should be noted. We were unable to use all five Waves of the BNLA study due to modal effects in the rates of psychological distress between face-to-face and telephone modes of data collection. Furthermore, we were unable to use any formal diagnostic measurement tools in the study and utilized the K6 which is a self-report measure of psychological distress that may be subject to recall bias and may also encourage socially desirable responses. Moreover, the K6 was not cross-culturally validated in all the languages used in the study. Lastly, use of single-item questions to assess social integration stressors precludes a more nuanced understanding regarding the severity of each stressor for each participant. Future research should assess post-migratory stressors using multi-dimensional and cross-culturally validated scales to better understand the effect of each stressor. Despite these limitations, this study’s strengths include its longitudinal and population-representative design with a community sample of humanitarian migrants, wide range of variables assessed and within-person analyses which allowed for a better understanding of individual trajectories of psychological distress over the 5-year follow-up period.

These findings have several implications for mental health clinicians, resettlement service providers and policymakers. It is important for mental health clinicians working with refugees to screen for trauma and regularly ask about various daily social integration and socioeconomic stressors, given these issues may be actively causing psychological distress. Refugee resettlement services should also provide opportunities for increased social connection, given social integration and social support have been found to be negatively associated with loneliness in the refugee population and may be used as a coping mechanism for resettlement-related stressors [44]. Service providers, as well as community organizations, need to continue addressing discrimination and implement programs that foster a stronger sense of belonging within the local community. One randomized-controlled trial found significant improvements in mental health following an intervention that integrated weekly meetings with a paired student partner where the refugee could practice English, exchange cultural knowledge with interpreters, and access another modality of social support over a 6-month period [10]. Another intervention found weekly, structured support groups with a peer/professional helper significantly improved perceived support, social integration and reduced loneliness post-intervention [45]. Active interventions as such would provide refugees with support at an individual and interpersonal level as well as resources to independently access other services [10]. Finally, policymakers and researchers should conduct pilot evaluations to assess the effectiveness of extending resettlement programmes beyond 18 months on post-migratory stressors and mental health outcomes, given current policies do not appear to be adequately supporting refugees in the early years of resettlement.

## Conclusion

Our study demonstrates the significant role that social integration stressors, particularly loneliness, have on the trajectories of psychological distress of refugees who have recently settled in Australia. The experience of pre- and peri-migratory traumatic events as well as socioeconomic stressors also continue to have a significant impact on psychological distress in the refugee population, highlighting the importance of trauma-informed practices and providing access to secure employment and housing. Our findings also highlight the need for further pilot studies to evaluate the effectiveness of extending refugee resettlement programmes beyond 18 months with a particular emphasis on interventions which facilitate social integration.

### Supplementary Information

Below is the link to the electronic supplementary material.

Supplementary file1 (DOCX 25 KB)

## Data Availability

The BNLA dataset used in the present study is publicly available to researchers who have obtained permission from the Australian Department of Social Services.

## Abbreviations

BNLA: Building a New Life in Australia
K6: Kessler Screening Scale for Psychological Distress
PA: Principal applicant
PTEs: Potentially traumatic events

## Footnotes

1 Using a total score range of 0 to 24, these cut offs are equivalent to 0 to 7 (low) and 8 and over (moderate/high).

2 *n* = 354 respondents are missing in this question as they did not complete an interview in Wave 3.
