# Supplementary material for: Trajectories of psychological distress and social integration in newly resettled refugees: findings from the Building a New Life in Australia longitudinal study
Source: Soc Psychiatry Psychiatr Epidemiol. 2023 Jul 1;59(8):1425–35. doi: 10.1007/s00127-023-02528-7 (PMC11291576; doi:10.1007/s00127-023-02528-7)
Supplement: Supplementary file 1 — Supplementary file1 (DOCX 25 KB) [file 127_2023_2528_MOESM1_ESM.docx]

**Title: Trajectories of psychological distress and social integration in newly resettled refugees: findings from the Building a New Life in Australia longitudinal study**

*Social Psychiatry and Psychiatric Epidemiology*

Thomas P. Nguyen^1,2,3^*, Shameran Slewa-Younan^1,2,4^, Pilar Rioseco^5,6^

1. Mental Health, School of Medicine, Western Sydney University, Sydney, Australia
2. Translational Health Research Institute, School of Medicine, Western Sydney University, Sydney, Australia.
3. Academic Unit of Child Psychiatry South West Sydney, School of Psychiatry, University of New South Wales, Sydney, Australia
4. Centre for Mental Health, Melbourne School of Population and Global Health, University of Melbourne, Melbourne, Victoria, Australia
5. Australian Institute of Family Studies, Melbourne, Australia
6. School of Public Health & Social Work, Queensland University of Technology, Brisbane, Australia

*Correspondence: tpnguyen99@gmail.com

Mental Health, School of Medicine, Western Sydney University, Locked Bag 1797, Penrith South DC NSW, Australia 1797

**Supplementary Table 1: Percentage of refugees experiencing moderate/high levels of psychological distress over time**

| Psychological distress categories (K6) | Wave 1 (2013/14) | | Wave 3 (2015/16) | | Wave 5 (2017/18) | |
| --- | --- | --- | --- | --- | --- | --- |
|  | N | % | N | % | N | % |
| Low (6-13) | 1,001 | 54.9 | 785 | 52.5 | 884 | 58.7 |
| Moderate/ high (14-30) | 822 | 45.1 | 711 | 47.5 | 621 | 41.3 |

Notes. K6 score in brackets. Includes all responding participants for each wave. Similar percentages were observed for the balanced sample: 47% at Wave 1, 48% at Wave 3, 41% at Wave 5, suggesting that attrition is not related to level of psychological distress in this sample.

**Supplementary Table 2: Percentage of participants with elevated psychological distress by baseline characteristics**

| Characteristic | N | % |
| --- | --- | --- |
| Sex |  |  |
| Male | 373 | 40.7 |
| Female | 449 | 49.6 |
| Location |  |  |
| Major city | 737 | 45.0 |
| Regional | 85 | 46.2 |
| Time since arrival |  |  |
| <6 months | 799 | 45.6 |
| 6-12 months | 23 | 32.4 |
| Region of birth |  |  |
| Middle East | 153 | 47.5 |
| Central Asia | 537 | 44.1 |
| Africa | 128 | 47.6 |
| South and South-East Asia | 153 | 47.5 |
| Pre-migration education |  |  |
| Never attended school | 153 | 47.5 |
| 6+ years of schooling | 537 | 44.1 |
| Post-school qualification | 128 | 47.6 |
| Number of PTE |  |  |
| 1 | 186 | 45.7 |
| 2-3 | 197 | 49.3 |
| 4+ | 183 | 50.8 |
| Does not apply | 62 | 38.0 |
| Prefer not to say | 39 | 40.6 |
| Unemployed household |  |  |
| Yes | 798 | 45.5 |
| No | 20 | 30.8 |
| Housing tenure |  |  |
| Temporary | 69 | 46.0 |
| Short term | 344 | 50.4 |
| Long term | 382 | 41.1 |
| Other | 12 | 42.9 |
| Experienced discrimination |  |  |
| No | 778 | 44.6 |
| Yes | 44 | 57.9 |
| Loneliness is source of stress |  |  |
| No | 615 | 41.8 |
| Yes | 190 | 62.5 |
| Waiting for family to migrate |  |  |
| No | 345 | 44.1 |
| Yes | 476 | 45.9 |
| Getting used to life in Australia is source of stress | |  |
| No | 545 | 40.9 |
| Yes | 260 | 58.7 |

Source. Analytical sample at Wave 1.

**Supplementary Table 3: Mean and standard deviation on baseline characteristics (continuous) by level of psychological distress**

| Characteristic | Low psychological distress | Moderate/high psychological distress |
| --- | --- | --- |
| Age (mean, SD) | 1.46 (0.50) | 1.55 (0.50) |
| Number of financial hardships (mean, SD) | 0.57 (0.98) | 0.99 (1.25) |
| English proficiency (mean, SD)^a^ | 2.07 (0.78) | 1.91 (0.75) |
| Sense of belonging (mean, SD)^b^ | 4.26 (0.95) | 3.85 (1.15) |

Source. Analytical sample at Wave 1.

**Supplementary Table 4: Sensitivity analysis of adjusted mixed-effects models predicting elevated psychological distress over time, excluding PTEs**

|  |  | | |
| --- | --- | --- | --- |
|  | Odds ratio | 95% CI  interval] | P>z |
| Time (ref. Wave 5, 2017/18) |  |  |  |
| Wave 1 (2013/14) | 0.67 | [0.54,0.84] | <0.001 |
| Wave 3 (2015/16) | 1.15 | [0.94,1.41] | 0.165 |
| **Demographic characteristics** |  | | |
| Age (cont.) | 1.02 | [1.01,1.02] | <0.001 |
| Female (ref. male) | 1.40 | [1.15,1.70] | <0.001 |
| Time since arrival (ref. <6 months) |  |  |  |
| 6-12 months | 0.64 | [0.39,1.04] | 0.070 |
| Region of birth (ref. Middle East) |  |  |  |
| Central Asia | 0.36 | [0.27,0.47] | <0.001 |
| Africa | 0.22 | [0.14,0.37] | <0.001 |
| South and South-East Asia | 0.24 | [0.17,0.34] | <0.001 |
| Education pre-migration (ref. post-school qualification) |  |  |  |
| No schooling | 1.00 | [0.67,1.50] | 0.984 |
| 6+ years of schooling | 1.04 | [0.79,1.38] | 0.768 |
| Lives in regional area (ref. major city) | 2.01 | [1.44,2.82] | <0.001 |
| **Socio-economic stressors** |  | | |
| Unemployed household (ref. household member in paid work) | 1.68 | [1.34,2.12] | <0.001 |
| Housing tenure (ref. long term housing) |  |  |  |
| Temporary | 1.33 | [0.95,1.86] | 0.093 |
| Short term | 1.38 | [1.10,1.72] | 0.005 |
| Other | 1.23 | [0.74,2.04] | 0.424 |
| Number of financial hardships (cont.) | 1.40 | [1.31,1.50] | <0.001 |
| **Social integration stressors** |  | | |
| English /proficiency (cont.) | 0.74 | [0.64,0.85] | <0.001 |
| Has experienced discrimination (ref. no) | 1.71 | [1.19,2.45] | 0.003 |
| Loneliness is a source of stress (ref. no) | 2.39 | [1.88,3.03] | <0.001 |
| Waiting for family to migrate (ref. no) | 1.24 | [1.04,1.47] | 0.015 |
| Sense of belonging (cont.) | 0.65 | [0.60,0.72] | <0.001 |
| Getting used to life in Australia is source of stress (ref. no) | 1.86 | [1.48,2.34] | <0.001 |
| Constant | 1.46 | [0.64,3.30] | 0.366 |
| Individual-level variance | 1.36 | [1.03,1.81] |  |

Notes. PTE: Potentially traumatic events. CI: confidence interval. OR: odds ratio. Ref: reference category. Cont: continuous.
